# 20(S)-Protopanaxadiol Inhibition of Progression and Growth of Castration-Resistant Prostate Cancer

**DOI:** 10.1371/journal.pone.0111201

**Published:** 2014-11-06

**Authors:** Bo Cao, Yanfeng Qi, Yan Yang, Xichun Liu, Duo Xu, Wei Guo, Yang Zhan, Zhenggang Xiong, Allen Zhang, Alun R. Wang, Xueqi Fu, Haitao Zhang, Lijing Zhao, Jingkai Gu, Yan Dong

**Affiliations:** 1 College of Life Sciences, Jilin University, Changchun, China; 2 Department of Structural and Cellular Biology, Tulane University School of Medicine, Tulane Cancer Center, New Orleans, Louisiana, United States of America; 3 Department of Pathology and Laboratory Medicine, Tulane University School of Medicine, Tulane Cancer Center, New Orleans, Louisiana, United States of America; 4 College of Basic Medical Sciences, Jilin University, Changchun, China; 5 National Engineering Laboratory for AIDS Vaccine, Jilin University, Changchun, China; Northwestern University, United States of America

## Abstract

Castration-resistant progression of prostate cancer after androgen deprivation therapies remains the most critical challenge in the clinical management of prostate cancer. Resurgent androgen receptor (AR) activity is an established driver of castration-resistant progression, and upregulation of the full-length AR (AR-FL) and constitutively-active AR splice variants (AR-Vs) has been implicated to contribute to the resurgent AR activity. We reported previously that ginsenoside 20(S)-protopanaxadiol-aglycone (PPD) can reduce the abundance of both AR-FL and AR-Vs. In the present study, we further showed that the effect of PPD on AR expression and target genes was independent of androgen. PPD treatment resulted in a suppression of ligand-independent AR transactivation. Moreover, PPD delayed castration-resistant regrowth of LNCaP xenograft tumors after androgen deprivation and inhibited the growth of castration-resistant 22Rv1 xenograft tumors with endogenous expression of AR-FL and AR-Vs. This was accompanied by a decline in serum prostate-specific antigen levels as well as a decrease in AR levels and mitoses in the tumors. Notably, the 22Rv1 xenograft tumors were resistant to growth inhibition by the next-generation anti-androgen enzalutamide. The present study represents the first to show the preclinical efficacy of PPD in inhibiting castration-resistant progression and growth of prostate cancer. The findings provide a rationale for further developing PPD or its analogues for prostate cancer therapy.

## Introduction

Androgen deprivation therapy, which disrupts androgen receptor (AR) signaling through castration or AR antagonists, is the first-line treatment for disseminated prostate cancer. However, progression to the presently incurable stage, termed castration-resistant prostate cancer (CRPC), invariably occurs [1]. Resurgent AR activity is an established driver of therapeutic failure and castration-resistant progression [2], [3]. Prostate cancer can adapt to androgen deprivation therapy by mutating AR, amplifying/overexpressing AR, upregulating constitutively-active AR splice variants (AR-Vs) that lack the ligand-binding domain, activating AR by androgen-independent mechanisms, and/or increasing intra-tumoral androgen levels through *de novo* androgen synthesis [4]–[17]. As a consequence, AR is reactivated in CRPC although the tumor is no longer responsive to androgen deprivation therapy.

Several new drugs targeting AR reactivation in CRPC have been developed, and two of these have been approved by the FDA for treatment of metastatic CRPC, *i.e.*, the androgen biosynthesis inhibitor abiraterone and the potent AR antagonist enzalutamide [18], [19]. They heralded a new era of prostate cancer therapy. However, many patients presented with therapy-resistant disease, and most initial responders developed resistance within months of therapy initiation, again accompanied by increased prostate-specific antigen (PSA), indicating reactivated AR signaling [18], [19]. One potential mechanism of resistance to abiraterone and enzalutamide has been ascribed to increased expression of the full-length AR (AR-FL) and AR-Vs [20]–[24]. Overexpression of AR-FL was shown to sensitize the receptor to low levels of androgen [25] and to convert prostate cancer growth from a castration-sensitive to a castration-resistant stage [10]. Increased expression of AR-Vs was shown to confer castration-resistant growth of prostate tumors [14], [15], [26]–[28], and to correlate with poor survival of CRPC patients [29]. Knocking down AR-FL or AR-Vs by shRNA in xenograft models can delay the progression of prostate cancer to castration resistance and/or suppress the growth of prostate tumor that has already progressed to the castration-resistant state [15], [30], [31]. Therefore, therapeutic approaches that can diminish the availability of both AR-FL and AR-Vs should offer considerable benefit in preventing and inhibiting prostate cancer recurrence after androgen deprivation therapy.

The ginseng root is one of the most commonly-used medicinal herbs in the Western world, particularly for cancer intervention [32]. Ginsenosides are considered the major pharmacologically-active ginseng constituents [33]. We previously reported that a main intestinal metabolite of ginsenosides, 20(S)-protopanaxadiol-aglycone (PPD), is effective in downregulating the expression and activity of AR, including both AR-FL and AR-Vs, in human prostate cancer cells [34]. The decrease in AR expression is due to PPD-mediated reduced transcription of the AR gene and increased proteasome-mediated degradation of AR-FL and AR-V proteins [34]. We further showed that PPD also inhibited AR expression in prostate xenograft tumors but not in the normal host prostates [34]. In the present study, we evaluated preclinically the potential of using PPD to improve the therapeutic outcome of androgen deprivation therapy.

## Materials and Methods

### Cell Lines and Reagents

LNCaP and 22Rv1 cells were obtained from American Type Culture Collection at Passage 4. CWR-R1 cells were provided by Dr. Elizabeth M. Wilson and cultured as described [35]. Cells used in this study were within 20 passages (∼3 months of non-continuous culturing). PPD was obtained from the Organic Chemistry Laboratory at Jilin University, Changchun, China. Compounds of purity of >98%, as determined by high-performance liquid chromatograph, were used in cell culture studies, and that of >95% was used in animal studies. Enzalutamide was purchased from Selleck Chemicals (Houston, TX), and the purity of >99% was confirmed by Nuclear Magnetic Resonance.

### Western Blotting

The procedure was described previously [36]. Immunoreactive bands were quantitated by densitometry and normalized to glyceraldehyde-3-phosphate dehydrogenase (GAPDH). The following antibodies were used: anti-GAPDH (Millipore), anti-AR (Millipore), and anti-AR-V7 (Precision Antibody).

### Quantitative Reverse Transcription-PCR (qRT-PCR)

qRT-PCR was performed as described [37]. The qPCR primer-probe sets for PSA, transmembrane protease, serine 2 (TMPRSS2), and cyclin A2 (CCNA2) were from IDT. The primer sequences for AR isoforms were as described [15].

### Reporter Gene Assay

The androgen-responsive element (ARE)-luciferase reporter plasmid, containing three repeats of the ARE region ligated in tandem to the firefly luciferase reporter [38], was used to measure AR *trans*-activating activity. It was transiently co-transfected into cells with the pRL-TK *Renilla* luciferase construct (Promega) at 20∶1 ratio as described [39]. Dual luciferase assay was conducted per manufacture's instruction (Promega), and the activity of the firefly luciferase was normalized to that of the *Renilla* luciferase.

### Tumor Xenograft Models

Male nude mice were obtained from the NCI Animal Production Center at 5–6 weeks of age. For the castration-resistant progression model of LNCaP tumors, mice were inoculated subcutaneously with 4×10^6^ LNCaP cells suspended in 50% Matrigel on the right flank after one week of adaptation. When the tumor size reached ∼100 mm^3^, castration was performed *via* a scrotal approach. The day following castration, the mice were randomly assigned to two groups and received 40 mg/kg PPD in olive oil or olive oil as control through oral gavage 6 days weekly. For the castration-resistant 22Rv1 tumor model, mice were surgically castrated after one week of adaptation, and allowed to recover for 3 days before they were inoculated subcutaneously with 4×10^6^ 22Rv1 cells on the right flank. The day following inoculation, the mice were randomized and placed on the same treatment regimens as described for the LNCaP model. The tumor dimensions and body weights were measured biweekly and weekly, respectively. Tumor volume was calculated as *0.524 x width^2^ x length* [40]. At the termination of the experiment, mice were anesthetized with Ketamine/Xylazine, blood collected for serum PSA determination using quantitative ELISA (United Biotech), and euthanized by CO_2_. Tumors were removed, weighed, and fixed in 10% formalin for paraffin embedding and histological analyses. All animal procedures were approved by the Tulane University Institutional Animal Care and Use Committee.

### AR Immunohistochemical Analyses

Immunohistochemistry was conducted as described previously [34]. As the negative control, the primary antibody was replaced with a non-immune IgG at the same concentration, and no reactivity was detected. Stained sections were scanned using the Aperio ScanScope Scanner, and digitalized images were analyzed with the Aperio WebScope-integrated algorithms. For AR staining, images were sampled sequentially throughout each section with areas of necrosis, preparation artifacts, and edges avoided. The intensity of nuclear staining was categorized to strong, weak, or negative as determined by the Aperio Nuclear V9 algorithm. Phospho-histone H3 staining was quantitated as described [41]. Five random microscopic fields were captured for each tumor section at 10x magnification, and the number of phospho-histone-H3-positive cells was manually counted.

### Statistical Analysis

The Student's two-tailed t test was used to determine the mean differences between treatment and control. Data are presented as mean ± SEM.

## Results

### PPD Downregulation of AR-FL and AR-Vs in Androgen-Deprived Condition

**Figure 1:**
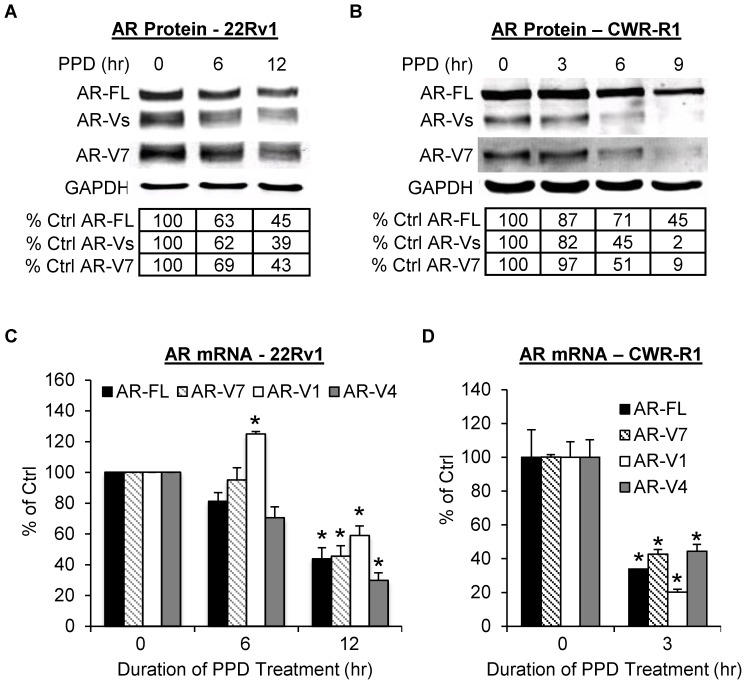
PPD downregulation of AR expression in androgen-deprived condition. **A & B.** Western blot analysis of AR proteins with an antibody recognizing all AR isoforms or specific for AR-V7 in 22Rv1 (A) or CWR-R1 cells (B). The numbers in the tables denote relative normalized intensities of the AR protein bands compared to the control value of 100. **C.** qRT-PCR analysis of the mRNA levels of AR-FL and AR-Vs in 22Rv1 cells. *, *P*<0.05 from control. Cells were cultured in androgen-deprived condition. PPD, 20 µg/ml. Error bars, SEM.

We first assessed the effect of PPD on AR-FL and AR-V protein levels in castration-resistant 22Rv1 and CWR-R1 cells cultured in androgen-deprived condition. These two cell models express AR-FL along with three ∼80-KDa major AR-Vs, namely AR-V7, AR-V1 (aka AR4), and AR-V4 (aka AR5) [15], [16]. Western blot analyses were conducted with an antibody recognizing all AR isoforms or specific for AR-V7. As the most abundant and active AR-V in the cells [15], AR-V7 is the only AR-V to which a specific antibody has been developed. As shown in Figures 1A and 1B, PPD downregulated both AR-FL and AR-Vs in a time-dependent manner, with the change in AR-Vs similar to that of AR-FL in 22Rv1 cells but slightly more significant than that of AR-FL in CWR-R1 cells. We then examined the effect of PPD on the mRNA levels of different AR isoforms in these cells cultured in androgen-deprived condition by qRT-PCR. As presented in Figures 1C and 1D, PPD treatment also reduced AR-FL and AR-V transcripts in both cell models. Interestingly, a modest but statistically significant increase of AR-V1 expression was observed at the early time point in 22Rv1 cells. However, the increase was not sustained with longer treatment. These results indicate that PPD can inhibit the expression of AR-FL and AR-Vs in androgen-deprived condition.

### PPD Suppression of Androgen-Independent AR Activity

**Figure 2:**
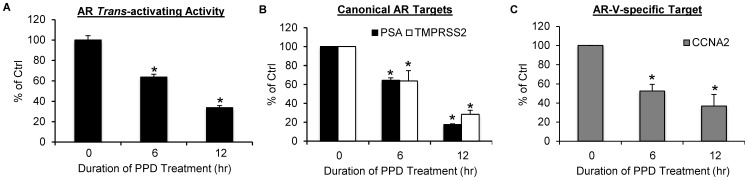
PPD suppression of androgen-independent AR transactivation. **A.** Reporter gene assay showing PPD inhibition of androgen-independent *trans*-activating activity of AR. 22Rv1 cells were co-transfected with the ARE-luciferase construct and the pRL-TK construct, and the activity of the firefly luciferase was normalized by that of the *Renilla* luciferase. **B & C.** qRT-PCR analyses showing PPD decrease the expression of the canonical AR targets PSA and TMPRSS2 (B) and the AR-V-specific target CCNA2 (C) in 22Rv1 cells. *, *P*<0.05 from control. Cells were cultured in androgen-deprived condition. PPD, 20 µg/ml. Error bars, SEM.

AR-Vs lack the ligand-binding domain and possess androgen-independent constitutive activity [15], [16], [42]. PPD downregulation of the AR-Vs would be expected to lead to suppression of androgen-independent AR transactivation. We hence assessed the effect of PPD on AR activity in 22Rv1 cells cultured in androgen-deprived condition by the reporter gene assay. As shown in Figure 2A, androgen-independent AR transactivation was depressed by PPD as a function of time. At 6 and 12 hr after PPD treatment, the activity was inhibited by 36% and 66%, respectively. We proceeded to examine the effect of PPD on endogenous AR-target genes, the canonical targets PSA and TMPRSS2 as well as the AR-V-specific target CCNA2 [20], [43], by qRT-PCR under the same condition. In line with the reporter gene result, all targets (Figures 2B & 2C) showed a time-dependent decrease of expression. Taken together, the data demonstrated the ability of PPD to inhibit androgen-independent AR-FL and AR-V activities.

### PPD Inhibition of Castration-Resistant Progression of LNCaP Tumors

We then evaluated the effect of PPD on prostate cancer recurrence after androgen deprivation therapy in the LNCaP xenograft model. LNCaP is an androgen-dependent human prostate cancer cell line and expresses predominantly AR-FL [44]. The development of LNCaP xenograft tumors requires androgens, and castration causes an initial decelerated growth of the xenografts [45], [46]. However, similar to human prostate cancers, the xenografts eventually become castration resistant and resume growth [45]–[49]. Male nude mice were implanted with LNCaP cells on the right dorsal flank. Castration was performed when the tumors reached ∼100 mm^3^. Castrated mice were then divided into two groups receiving either olive oil as control or 40 mg/kg PPD through oral gavage 6 days per week. Treatment was initiated the day following castration.

**Figure 3:**
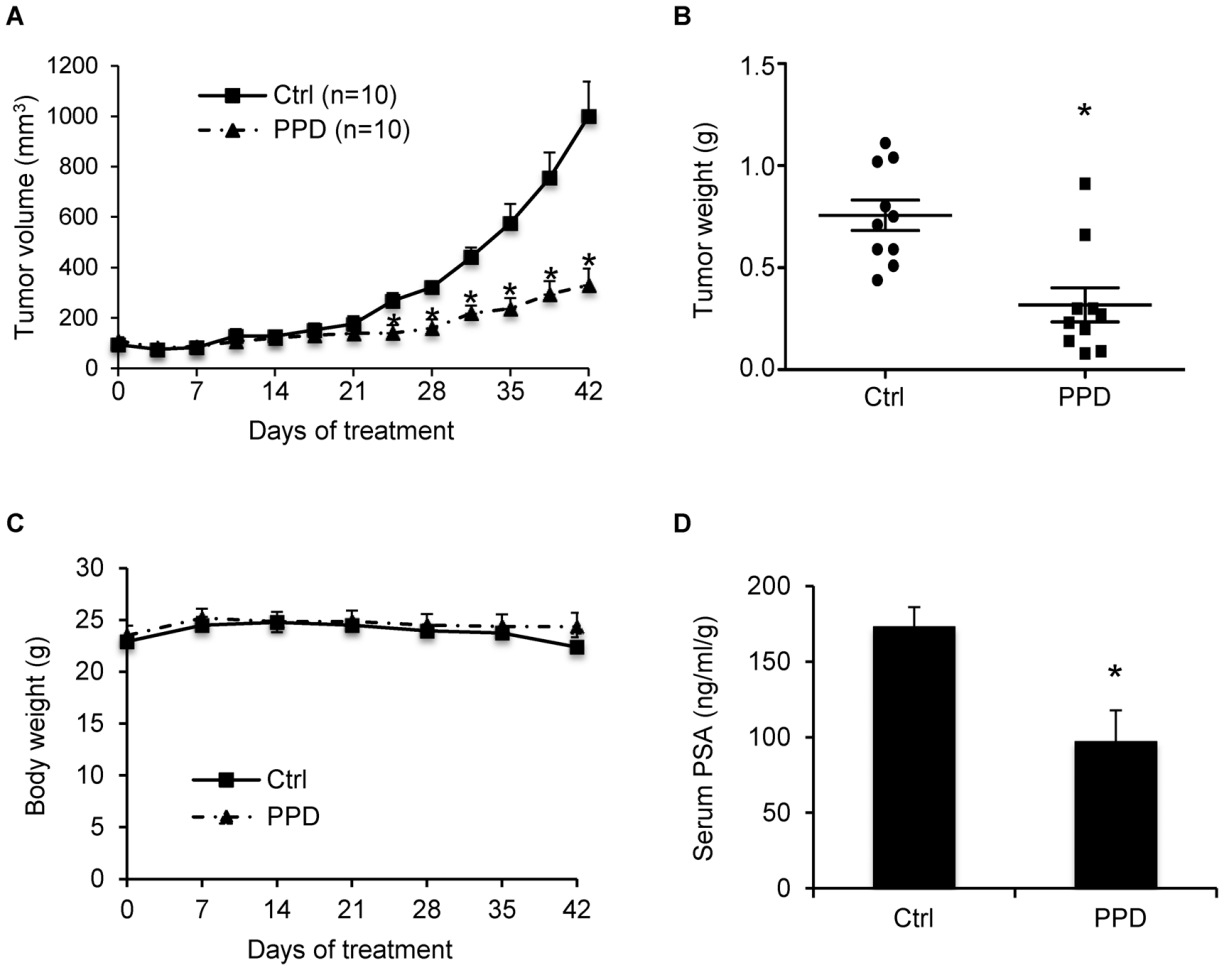
PPD inhibition of castration-resistant progression of LNCaP xenograft tumors. LNCaP cells were inoculated into gonad-intact nude mice, and surgical castration was performed when the tumors reached ∼100 mm^3^. Treatment with 40 mg/kg PPD through oral gavage 6 days per week was initiated the day following castration (n = 10). **A.** Mean tumor volumes. **B.** Individual tumor weights at the conclusion of the experiment. **C.** Mean mouse body weights. **D.** Mean serum PSA level determined by ELISA, normalized by tumor weights, at the conclusion of the study. *, *P*<0.05 from the control group. Error bars, SEM.

Compared to LNCaP tumors grown in gonad-intact mice [45], [46], there was a deceleration of tumor growth in both groups during the first two weeks of treatment as an initial response to castration (Figure 3A). The tumors in the control group resumed growth starting from day 17 of treatment as a result of acquiring castration resistance, whereas the tumors in the PPD group remained small. From day 25, the difference in the average tumor size between the two groups became statistically significant. At the termination of the experiment, the average tumor size was 1000 mm^3^ in the control group, but 330 mm^3^ in the PPD group, indicating ∼67% inhibition of tumor regrowth by PPD. In line with this result, the average tumor weights were 0.76 g and 0.32 g in control and PPD groups, respectively (Figure 3B). During the 42 days period of treatment, PPD did not appear to cause toxicity in the mice as indicated by no decrease in body weight compared to the control group (Figure 3C).

**Figure 4:**
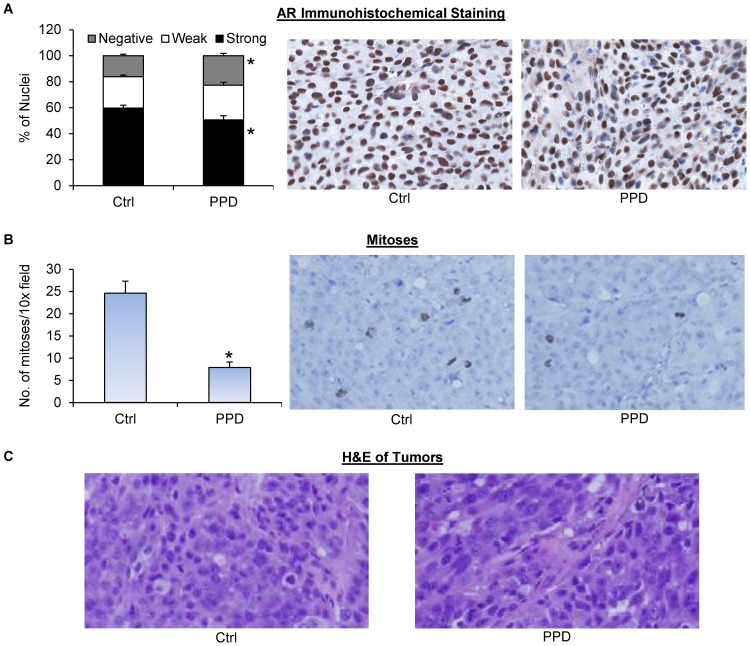
PPD inhibition of AR expression and mitosis in castration-resistant LNCaP xenograft tumors. Tumors were harvested at the end of the experiment of Figure 3. **A.** AR immunohistochemical staining of the tumor sections. Left panel, quantitation of the data. Right panels, representative images. **B.** Phospho-histone H3 immunohistochemical staining of the tumor sections. Left panel, quantitation of the data. Right panels, representative images. **C.** H&E staining of the tumor sections. *, *P*<0.05 from the control group. Error bars, SEM.

Prostate tumor relapse after castration is associated with elevated serum PSA levels [50]. We measured the levels of PSA in mouse serum using ELISA. As shown in Figure 3D, PPD supplementation led to an almost 50% drop in mean serum PSA level, from 173 ng/ml/g in the control group to 97 ng/ml/g in the PPD group. The levels were normalized by tumor weight. Thus, the drop was not a consequence of tumor growth inhibition, but rather an indication of PPD suppression of AR reactivation in the tumors. We then measured AR protein expression by immunohistochemistry in formalin-fixed tissues. As shown in Figure 4A, PPD treatment decreased the percentage of cells with strong AR staining while increasing the percentage of cells with negative staining. The data therefore corroborated our *in vitro* results, showing the effectiveness of PPD in downregulating AR level and activity in CRPC cells *in vivo*.

We further evaluated the effect of PPD on mitosis in the tumors via immunohistochemistry using an antibody against phospho-histone H3, a marker of mitosis [41]. As shown in Figure 4B, PPD supplementation caused a significant decrease in the mean number of mitoses per image field. Hematoxylin and eosin (H&E) staining of the tissues showed no apparent histopathological change of the tumors after PPD treatment (Figure 4C). Collectively, the data suggest the potential of using PPD to prevent prostate cancer relapse after androgen deprivation therapy.

### PPD Inhibition of the Growth of Castration-Resistant 22Rv1 Xenograft Tumors

**Figure 5:**
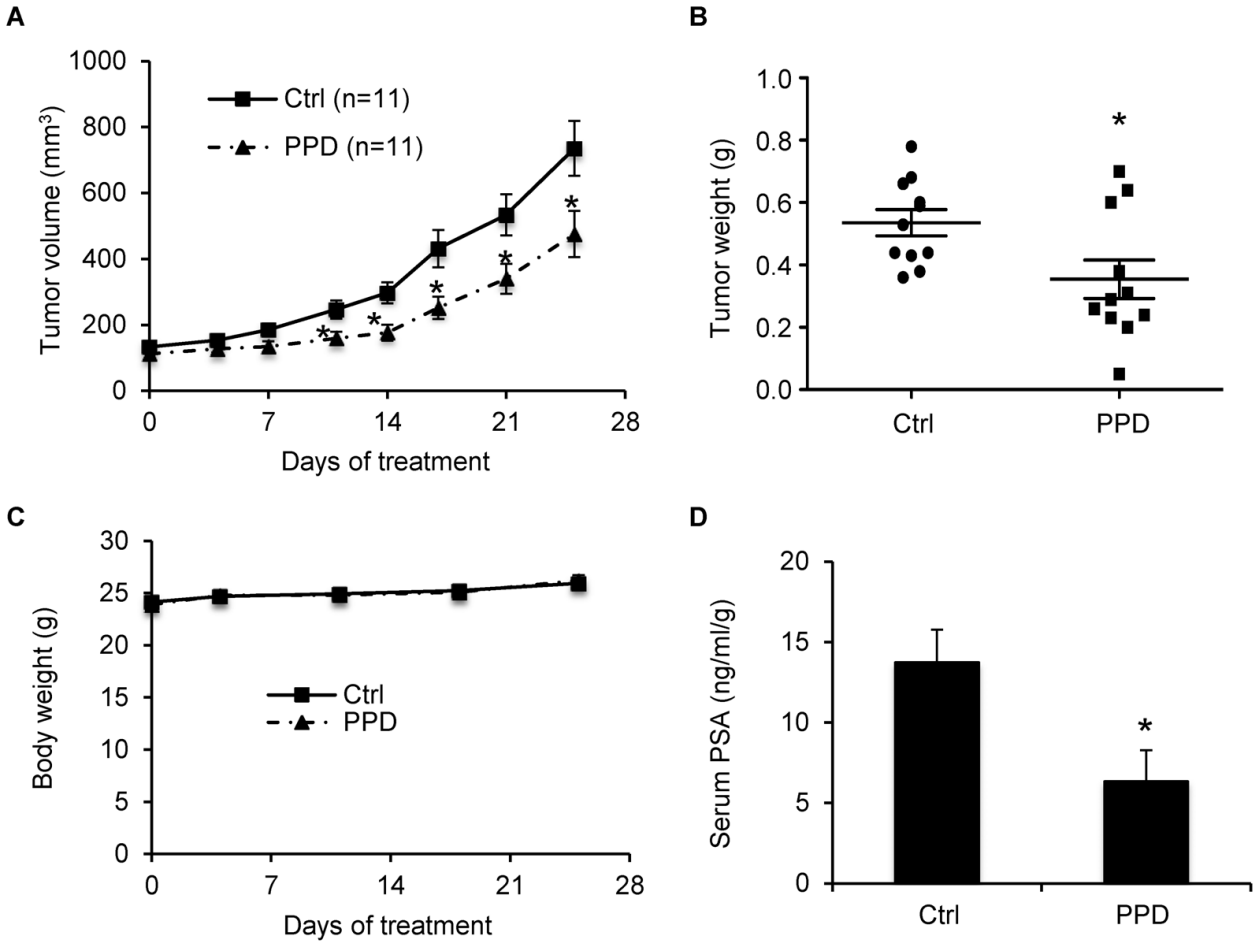
PPD inhibition of the growth of castration-resistant 22Rv1 xenograft tumors. 22Rv1 cells were inoculated into castrated nude mice. When the tumors reached ∼100 mm^3^, the mice were treated with 40 mg/kg PPD through oral gavage 6 days per week (n = 11). **A.** Mean tumor volumes. **B.** Individual tumor weight at the conclusion of the experiment. **C.** Mean mouse body weights. **D.** Mean serum PSA level determined by ELISA, normalized by tumor weights, at the conclusion of the study. *, *P*<0.05 from the control group. Error bars, SEM.

**Figure 6:**
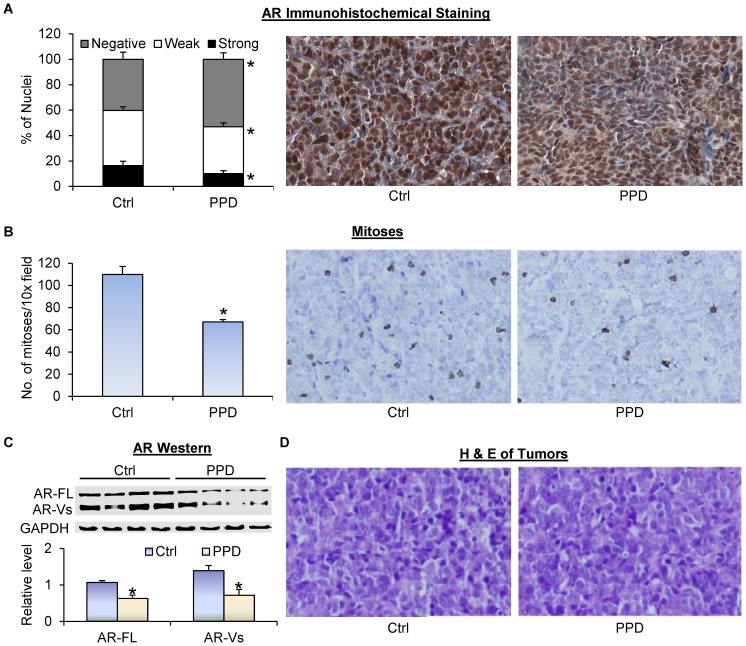
PPD inhibition of AR expression and mitosis in castration-resistant 22Rv1 xenograft tumors. Tumors were harvested at the end of the experiment of Figure 5. **A.** AR immunohistochemical staining of the tumor sections. Left panel, quantitation of the data. Right panels, representative images. **B.** Phospho-histone H3 immunohistochemical staining of the tumor sections. Left panel, quantitation of the data. Right panels, representative images. **C.** Western blot analysis of the tumors using a pan-AR antibody. Quantitation of the data was presented below the blots. **D.** H&E staining of the tumor sections. *, *P*<0.05 from the control group. Error bars, SEM.

We next assessed the ability of PPD, in comparison with the next-generation antiandrogen enzalutamide, to inhibit the growth of prostate tumors that are already castration resistant by using the 22Rv1 xenograft model. 22Rv1 cells were inoculated into the right dorsal flank of castrated male nude mice. Administration of 40 mg/kg PPD or 10 or 30 mg/kg enzalutamide through oral gavage 6 days per week was initiated when the tumors reach ∼100 mm^3^. As shown in Figure 5A, PPD significantly inhibited the growth of the 22Rv1 tumors. At the conclusion of the experiment, the average weight of the tumors was 0.35 g in the PPD group, but 0.54 g in the control group, indicating ∼35% inhibition of tumor growth by PPD (Figure 5B). This was also associated with ∼50% decline in serum PSA levels (Figure 5D) as well as a significant decrease in AR immunostaining signal detected using a pan-AR antibody (Figure 6A) and the number of mitoses (Figure 6B) in the tumors. Western blot analysis further showed PPD downregulation of both AR-FL and AR-Vs in the tumors (Figure 6C). PPD did not cause decrease in the body weights of the mice (Figure 5C) or apparent histopathological change of the tumors (Figure 6D).

**Figure 7:**
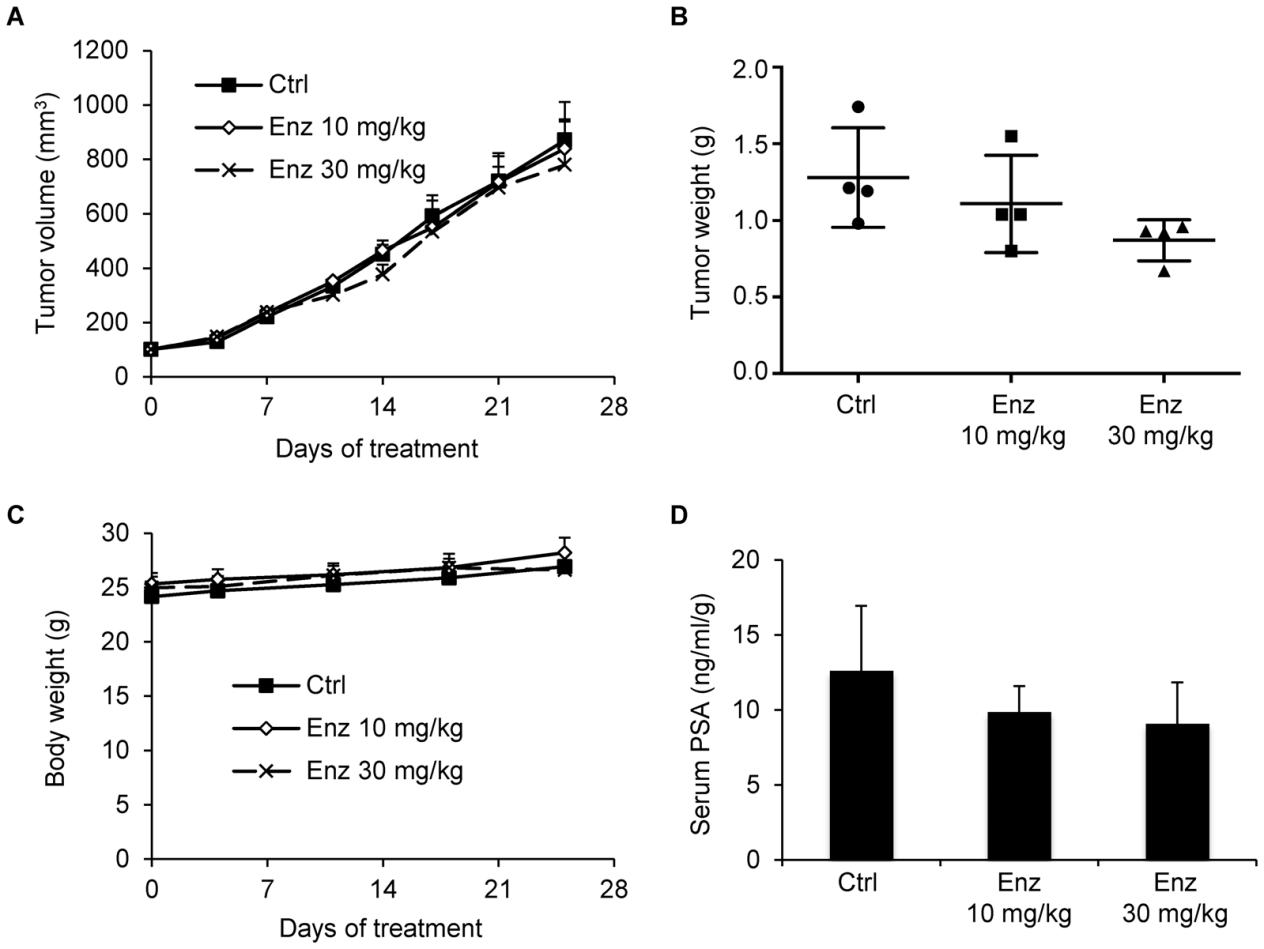
Enzalutamide did not affect the growth of castration-resistant 22Rv1 xenograft tumors. 22Rv1 cells were inoculated into castrated nude mice. When the tumors reached ∼100 mm^3^, the mice were treated with 10 or 30 mg/kg enzalutamide through oral gavage 6 days per week (n = 4). **A.** Mean tumor volumes. **B.** Individual tumor weight at the conclusion of the experiment. **C.** Mean mouse body weights. **D.** Mean serum PSA level determined by ELISA, normalized by tumor weights, at the conclusion of the study. Enz, enzalutamide. Neither of the treatment groups showed statistical difference from the control group in any of the endpoints. Error bars, SEM.

**Figure 8:**
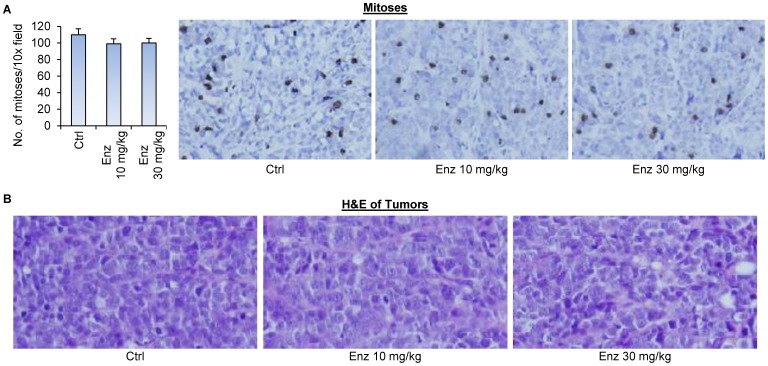
Enzalutamide did not inhibit mitosis in castration-resistant 22Rv1 xenograft tumors. Tumors were harvested at the end of the experiment of Figure 7. **A.** Phospho-histone H3 immunohistochemical staining of the tumor sections. Left panel, quantitation of the data. Right panels, representative images. **B.** H&E staining of the tumor sections. Enz, enzalutamide. Error bars, SEM.

Interestingly, enzalutamide, at neither dose, was able to inhibit the growth of 22Rv1 tumors (Figure 7A), suppress serum PSA levels in the mice (Figure 7D), inhibit mitosis (Figure 8A), or cause apparent histopathological change of the 22Rv1 tumors (Figure 8B). At the conclusion of the experiment, there was a trend of decrease in tumor weights with increasing dose of enzalutamide (Figure 7B). However, the decrease was not statistically significant, possibly due to the small sample size of the experiment. Nonetheless, the decrease observed with 10 mg/kg enzalutamide, the most commonly-used dose of enzalutamide that is effective against castration-resistant LNCaP xenograft models [27], [51], [52], was rather marginal. Taken together, the data suggest the potential of using PPD to inhibit the growth of CRPC.

## Discussion

We demonstrated previously that PPD suppresses androgen signaling through decreasing the expression of both AR-FL and AR-Vs [34]. In the present study, we extended the observations to androgen-deprived condition, showing androgen-independent downregulation of AR expression and transactivation by PPD. More importantly, the present study demonstrated, for the first time, the potential of using PPD to improve the therapeutic outcome of androgen deprivation therapy. We showed, in xenograft models, that PPD prevents or delays the development of CRPC after androgen deprivation and inhibits the growth of castration-resistant prostate tumors with endogenous expression of AR-FL and AR-Vs.

Rise in serum PSA level is used as a marker for biochemical recurrence after therapy. We showed that PPD supplementation leads to a reduction in serum PSA levels. Moreover, the reduction is not simply a consequence of decrease of tumor sizes by PPD, since the effect remains significant after normalization by tumor weight. The data, instead, indicate the ability of PPD to suppress AR reactivation in tumors relapsed after androgen deprivation and in tumors that are castration resistant, therefore providing a mechanistic basis for the ability of PPD to inhibit castration resistance.

The dose of PPD that was used in our xenograft studies was 40 mg per kg of mouse body weight. Based on the body surface area conversion [53], the human equivalent dose of 40 mg/kg PPD is 3.24 mg/kg. This equates to a daily dose of 194 mg for a 60-kg adult. Daily supplementation of 2 g of ginseng extract to adults with an average weight of 60 kg showed no adverse effect in human clinical trials [54], [55]. Since the ginsenosides content of the extract was 14.5% [54], the daily dose of ginsenosides in these trials was 290 mg. Thus, the dose that we used in our animal studies is achievable in human.

Despite the long appreciation of the importance of targeting AR signaling for prostate cancer treatment, no therapy has been developed to date to target AR directly to reduce its availability. In addition to PPD, several other compounds have been shown pre-clinically to reduce the levels of AR-FL and AR-Vs and to inhibit the growth of CRPC cells [26], [56]–[61]. These compounds may serve as an effective antidote to overcoming resistance to androgen deprivation therapy, particularly for resistance due to upregulated expression of AR-Vs. Because of the lack of the ligand-binding domain, AR-Vs cannot be targeted by current androgen deprivation therapies, including the new drugs abiraterone and enzalutamide. This is reflected by our data showing the ineffectiveness of enzalutamide against the growth of AR-V-expressing 22Rv1 xenograft tumors in castrated host. On the other hand, the growth of these tumors can be inhibited by PPD at a pharmacologically achievable dose. These findings support the potential of using PPD in combination with these new drugs for prostate cancer therapy. Determining the combinatory efficacies and the best sequences of treatments is an area of our ongoing research. Taken together, the findings from the current study not only provide a rationale for further developing PPD or its analogue for intervention of CRPC, but also substantiate reducing AR-FL and AR-V availability as a viable approach to improve therapeutic outcome of androgen deprivation therapy.
